# Morphology of the distal thoracic duct and the right lymphatic duct in different head and neck pathologies: an imaging based study

**DOI:** 10.1186/s13005-016-0108-y

**Published:** 2016-03-31

**Authors:** Ferdinand J. Kammerer, Benedikt Schlude, Michael A. Kuefner, Philipp Schlechtweg, Matthias Hammon, Michael Uder, Siegfried A. Schwab

**Affiliations:** Institute of Radiology, University Erlangen-Nuremberg, Maximiliansplatz 1, D-91054 Erlangen, Germany

**Keywords:** Head and neck pathologies, Distal thoracic duct, Right lymphatic duct, Computed tomography

## Abstract

**Background:**

The purpose of this study was to assess the influence of head and neck pathologies on the detection rate, configuration and diameter of the thoracic duct (TD) and right lymphatic duct (RLD) in computed tomography (CT) of the head and neck.

**Methods:**

One hundred ninety-seven patients were divided into the subgroups "healthy", "benign disease" and "malignant disease". The interpretation of the images was performed at a slice thickness of 3 mm in the axial and coronal plane. In each case we looked for the distal part of the TD and RLD respectively and subsequently evaluated their configuration (tubular, sacciform, dendritic) as well as their maximum diameter and correlated the results with age, gender and diagnosis group.

**Results:**

The detection rate in the study population was 81.2 % for the TD and 64.2 % for the RLD and did not differ significantly in any of the subgroups. The predominant configuration was tubular. The configuration distribution did not differ significantly between the diagnosis groups. The mean diameter of the TD was 4.79 ± 2.41 mm and that of the RLD was 3.98 ± 1.96 mm. No significant influence of a diagnosis on the diameter could be determined.

**Conclusions:**

There is no significant influence of head/neck pathologies on the CT detection rate, morphology or size of the TD and RLD. However our study emphasizes that both the RLD and the TD are detectable in the majority of routine head and neck CTs and therefore reading physicians and radiologists should be familiar with their various imaging appearances.

## Background

Computed tomography (CT) is a frequently used imaging method for pathologies of the head and neck, which represents a quite complex anatomic region. Various anatomic structures such as arteries or veins have to be differentiated and assessed by the reading physician, for both pure diagnostic purposes and/or therapy planning. As a CT of the head and neck region usually encloses parts of the upper mediastinum, both the distal thoracic duct (TD) and the right lymphatic duct (RLD) are included in the examinations as well and are detectable by CT [1]. The function of these main lymphatic vessels is to drain the whole body’s lymphatic fluid into the venous system: the right side of the thorax, the right arm and the right side of the head and neck are drained by the RLD into the junction of the right internal jugular and the right subclavian vein. The TD drains the rest of the body into the junction of the left internal jugular and the left subclavian vein.

While there are various reports in the literature about the influence of pathologies of the body trunk (e.g. malignancies, portal venous hypertension, congestive heart failure) on the imaging morphology and function of the main lymphatic vessels [2–9] to the best of our knowledge there exist no data regarding diseases of the head and neck. Therefore the aim of this study was to assess the influence of head and neck pathologies on the morphology of the distal thoracic duct and right lymphatic duct in CT.

## Methods

### Patients and diagnoses

The study was conducted in accordance with the guidelines of the Declaration of Helsinki and with approval of the ethics committee of the University of Erlangen-Nuremberg (23-06-2015). Written informed consent had been obtained from all patients. Our RIS (radiology information system) was searched for patients who underwent routine CT of the head and neck with intravenous contrast media (CM) application in a retrospective period of 30 months. In order to keep the possibility of any iatrogenic influence on the ducts' morphology as low as possible patients with a history of cervical surgery or radiation and any history of systemic chemotherapy were excluded from evaluation. To asses a potential influence of patient’s age on the CT morphology of the RLD and TD two age groups were defined: Age group A consisted of patients ≤ 50 years, age group B of patients > 50 years. Regarding the head and neck diagnoses the patients were divided into three particular diagnosis groups: Diagnosis group 1 included healthy patients without any visible disease on the CT scans. Diagnosis group 2 consisted of patients with benign and diagnosis group 3 of patients with malignant disease. If a pathology was present, its side of the neck was noted, pathologies crossing or reaching the midline were considered as both sided. All malignant lesions were pathologically proven (either before or after the CT scan). Benign lesions were either proven clinically, pathologically or by follow up examinations.

### Imaging and reading technique

To avoid any technical bias resulting from different CT technologies only patients examined on one particular scanner at our institution (Somatom Sensation 64, Siemens Healthcare, Erlangen, Germany) were included. Scanning parameters (120 kV, 200 eff. mAs, anatomy based tube current modulation) were identical in all examinations. The collimation was 64 x 0.6 mm. Patients were examined in the supine position with their arms lowered. Intravenous contrast media (Imeron 350, Bracco Imaging, Konstanz, Germany) was applied via an antecubital vein at a flow rate of 2.5 ml/s and the scan was started after a delay of 80 seconds.

All examinations were evaluated on a dedicated PACS (Picture Archiving and Communication System) workstation (syngo.plaza, Siemens, Germany). All examinations were anonymized and randomized. A board-certified radiologist (Siegfried A. Schwab) and a Ph.D. student (Benedikt Schlude) evaluated the distal TD and RLD in consensus on axial and coronal 3 mm reconstructions in soft tissue windowing (window 400, center 50).

### Evaluation

If both sides of the lower neck were not readable because of artifacts (e.g. due to pacemaker material or retained, highly concentrated intravenous CM), the examination was excluded from our evaluation. On the other hand if only one side of the lower neck was unreadable due to artifacts, the other side was included into the evaluation anyway.

Concerning the main lymphatic vessels the readers at first assessed if the RLD and TD could be identified at all. Criterion of identifiability was the visualization of a continuous non venous, non arterial vessel draining into the venous system on either side. In case of the TD there had to be a visible continuity into the upper mediastinum. The RLD or TD were considered as not detectable if their side of the neck was readable but no vessel meeting the criteria of identification was detected.

**Fig. 1:**
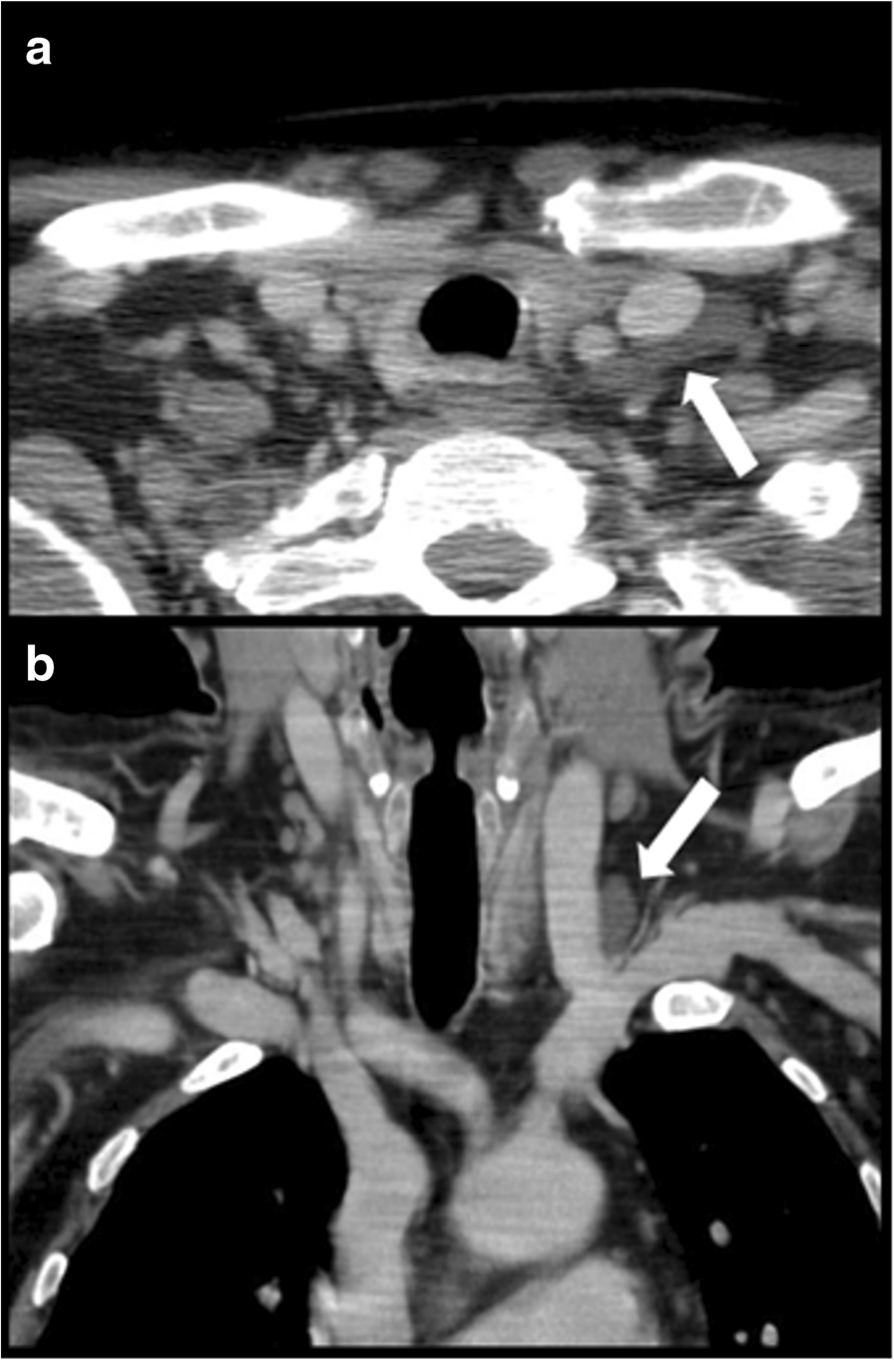
A 66 year old male with pharyngeal lymphoma. Contrast enhanced computed tomography in the axial (**a**) and coronal (**b**) plane. Tubular (axial plane) configuration of the thoracic duct (arrows)

**Fig. 2:**
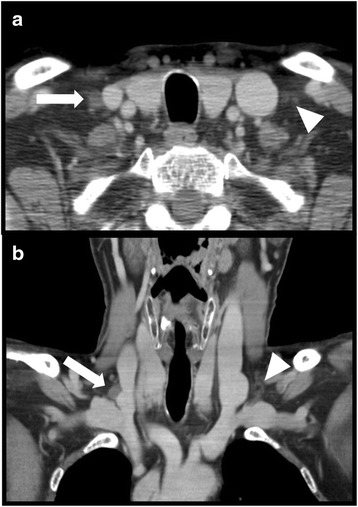
A 55 year old male with laryngeal carcinoma. Contrast enhanced computed tomography in the axial (**a**) and coronal (**b**) plane. Sacciform configuration of the right lymphatic duct in both planes (arrows). The thoracic duct shows a dendritic morphology (arrowheads)

**Fig. 3:**
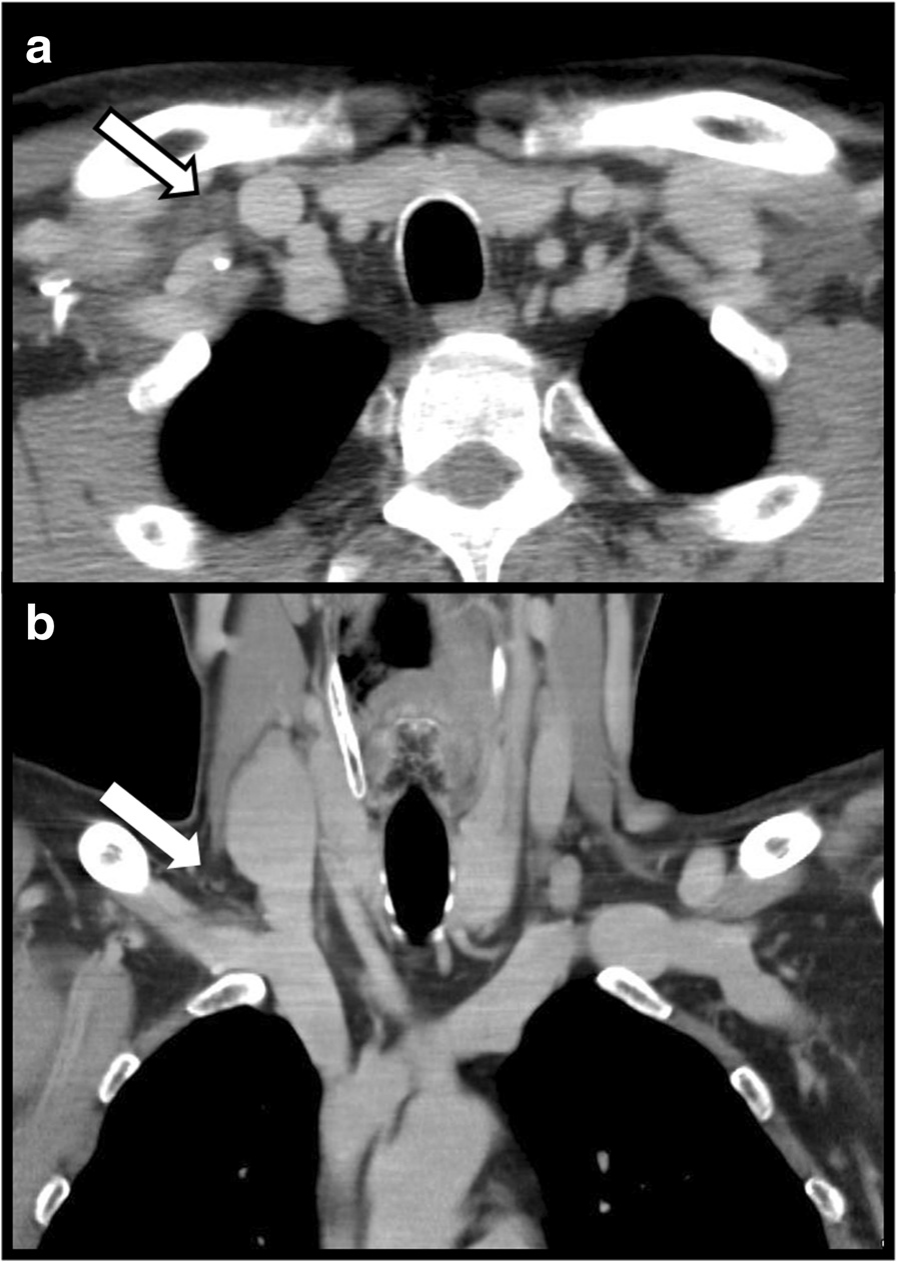
A 54 year old male with cancer of the tongue. Contrast enhanced computed tomography in the axial (**a**) and coronal (**b**) plane. Dendritic configuration of the right lymphatic duct (arrows)

If the main lymphatic vessels could be identified, their configuration was classified as either tubular, dendritic or sacciform: A tubular classification was chosen, when the vessel was mainly cylindrical in its morphology (Fig. 1). In case of a circumferential convex border of the distal lymphatic vessel, its configuration was considered as sacciform (Fig. 2). A dendritic configuration was considered at the presence of multiple smaller lymphatic vessels conjoining just before the drainage into the veins (Figs. 2 and 3). Moreover the largest transverse diameter of each identifiable main lymphatic vessel was measured and noted. In the dendritic configuration group the overall diameter of the area of confluence of the particular smaller vessels just before draining into the venous system was measured.

**Fig. 4:**
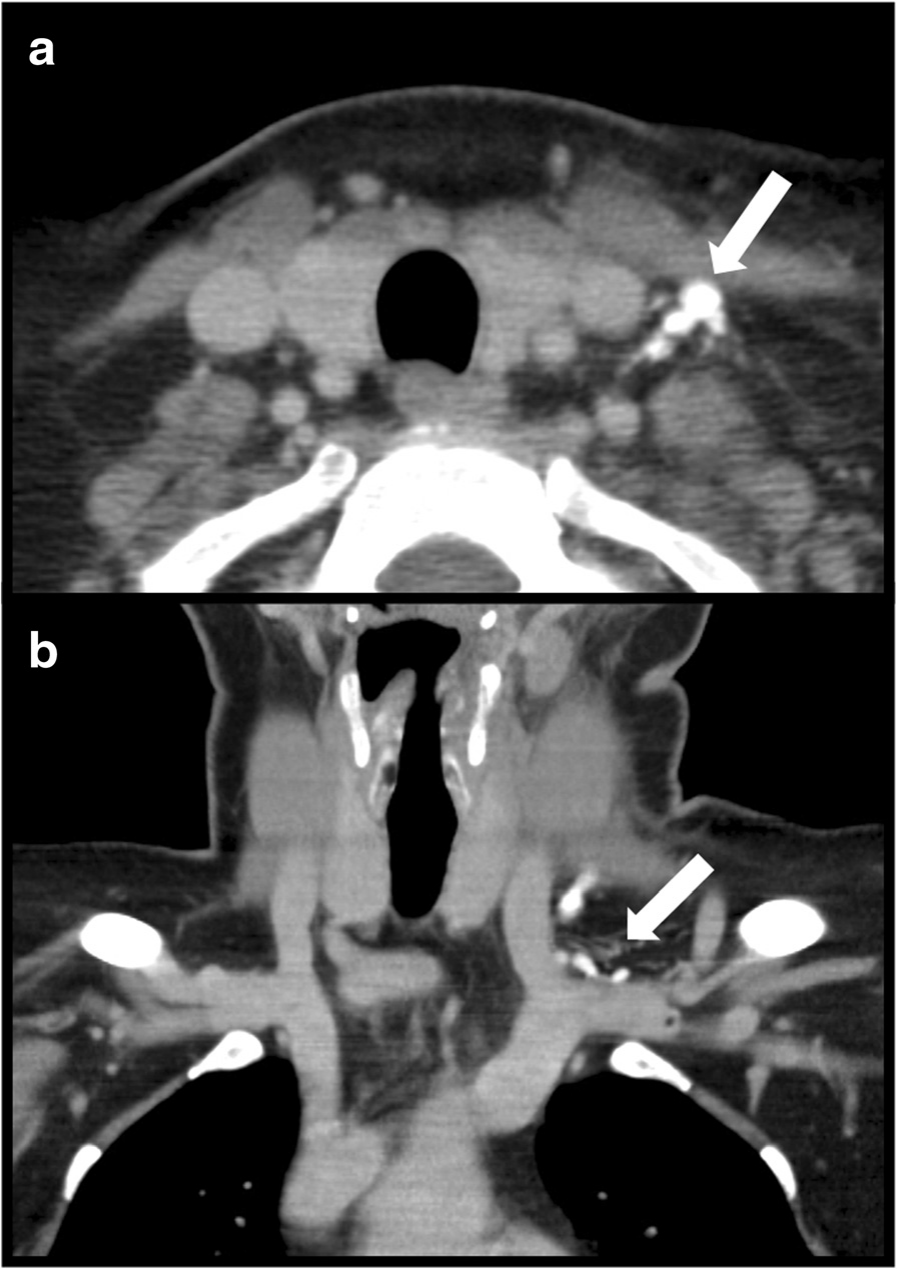
A 57 year old female with parotitis. Contrast enhanced computed tomography in the axial (**a**) and coronal (**b**) plane. Reflux of contrast media into a dendritic configured thoracic duct (arrows)

During the evaluation in some RLDs and TDs the readers observed very high Hounsfield Units (Fig. 4), which was considered a reflux of CM from the venous into the lymphatic vessel [1, 10]. The presence of this phenomenon was noted for both sides.

### Statistical analysis

All statistical evaluations were performed using dedicated software (SAS-Analyst Version 9.2, SAS Institute, USA). Data concerning the detectability of the lymphatic vessels, their diameters and configurations in various subsets of the study population were compared and checked for differences between the diagnosis groups (no pathology, benign pathology, malignant pathology). As both main ducts predominantly drain the lymph of either the right (RLD) or the left (TD) side of the neck, a differentiation between the sides of the pathology regarding the evaluated duct was made. The pathology was considered as ipsilateral if the pathology was on the particular duct’s drainage side and as contralateral if it was on the opposite side of the neck. Furthermore for the analysis of the vessels’ diameters the independent variables age (>50 years, ≤ 50 years) and gender (female, male) were included in the analysis. As statistical tests were chosen: T-Test, Kruskal-Wallis-Test, Wilcoxon-Test and Chi-Quadrat-Test. *P*-values < 0.05 were considered significant. Due to the lack of knowledge about the laterality of the injection site of the CM, the frequency of CM reflux into a lymph vessel could not be analyzed statistically.

## Results

Within time period of retrospective analysis 457 CTs of the neck were identified. Due to a history of surgery, radiotherapy or chemotherapy or the situation of both sided artifacts 260 examinations had to be excluded from the analysis. Therefore 197 examinations were included into the evaluation. 131 patients (66.5 %) were male (m), 66 (33.5 %) were female (f). Age was between 12 and 89 years (mean 59.0 years). Number of patients ≤ 50 years (age group A) was 49, whereas it was 148 for patients > 50 years (age group B). Diagnosis group 1 (no pathology) consisted of 32 patients (16.2 %; 14 m, 18 f). Diagnosis group 2 (benign pathology) consisted of 47 patients (23.9 %; 20 m, 27 f) and the pathologies noted in this group were: inflammatory/infectious (*n* = 25), benign tumor (*n* = 10), osteonecrosis (*n* = 5), developmental/other (*n* = 7). Diagnosis group 3 (malignant pathology) consisted of 118 individuals (59.9 %; 97 m, 21 f) and the pathologies in this group were: carcinoma (*n* = 111), lymphoma (*n* = 4), sarcoma (*n* = 2) and melanoma (*n* = 1). The benign pathology was right sided in 21, left sided in 15 and on both sides in 11 cases, the numbers for malignant disease were 45, 37 and 36 respectively. While the examinations could be evaluated on the left side of the neck for all 197 patients, this was the case in only 187 patients on the right side due to artifacts.

### Detectability of the TD and RLD

The TD could be identified in 160/197 (81.2 %) of the patients; the detectability rate was 87.5 % in diagnosis group 1, 78.8 % in group 2 and 80.5 % in group 3. This difference was not significant (*p* = 0.59). No significant difference between the diagnosis groups for ipsilateral pathologies could be observed (group 2 84.6 %; group 3 76.7 %; *p* = 0.37). Regarding the patients' age the detectability rate was 75.5 % in age group A and 83.1 % in group B (*p* = 0.24). In men the TD could be identified in 84.7 % and in women in 83.3 % (*p* = 0.16).

The RLD could be identified in 120/187 (64.2 %) of the patients; the detectability rate was 61.3 % in diagnosis group 1, 65.1 % in group 2 and 64.6 % in group 3. This difference was not significant (*p* = 0.93). Also no significant difference between the diagnosis groups for ipsilateral pathologies could be observed (group 2 67.9 %; group 3 64.9 %; *p* = 0.87). Regarding the patients' age the detectability rate was 63.6 % in age group A and 64.3 % in group B (*p* = 0.93). In men the RLD could be identified in 66.4 % and in women in 59.3 % (*p* = 0.35).

### Configuration of the TD and RLD

The configuration of the TD was tubular in 108 (67.5 %), sacciform in 34 (21.3 %) and dendritic in 18 (11.3 %) of all examinations. The distribution was 64.3 %, 21.4 % and 14.3 % for diagnosis group 1, 67.6 %, 27.0 % and 5.4 % in diagnosis group 2 and 68.4 %, 18.9 % and 12.6 % in diagnosis group 3. The distribution of configurations did not differ significantly between the diagnosis groups, no matter if the side of pathology was taken into account (*p* = 0.54) or not (*p* = 0.67). There was no significant influence of the gender concerning the configuration (*p* = 0.65). However a significant difference in configurations between age groups was found: patients > 50 years showed tubular or dendritic TDs more often than individuals ≤ 50 years who had more sacciform TDs (*p* = 0.02).

The configuration of the RLD was tubular in 53 (44.2 %), sacciform in 23 (19.2 %) and dendritic in 44 (36.7 %) of all evaluable examinations. The distribution was 47.4 %, 10.5 % and 42.1 % for diagnosis group 1, 42.9 %, 17.9 % and 39.3 % in diagnosis group 2 and 43.8 %, 21.9 % and 34.2 % in diagnosis group 3. The distribution of configurations did not differ significantly between the diagnosis groups, no matter if the side of pathology was taken into account (*p* = 0.82) or not (*p* = 0.84). Also there was no significant influence of the gender (*p* = 0.83) or age group (*p* = 0.10).

### Diameter of the TD and RLD

**Fig. 5:**
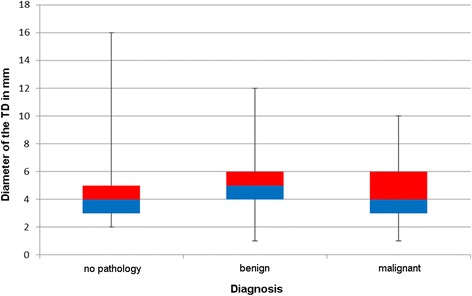
Box-Whisker-Plot for the diameter of the thoracic duct (TD) depending on diagnosis

In all TDs the mean diameter was 4.79 ± 2.41 mm. Males had a mean diameter of 4.96 ± 2.49 mm while the TD in women measured 4.4 ± 2.19 mm. The difference was not significant (*p* = 0.15). Healthy patients (group 1) had a mean diameter of 4.54 ± 2.78 mm, whereas it was 5.08 ± 2.37 mm in group 2 and 4.75 ± 2.32 mm in group 3 (Fig. 5). Taking into account only ipsilateral pathologies the mean diameter in group 2 was 4.55 ± 2.02 mm and 4.54 ± 2.24 mm in group 3. Neither of these differences were significant (*p* = 0.4 and *p* = 0.88).

**Fig. 6:**
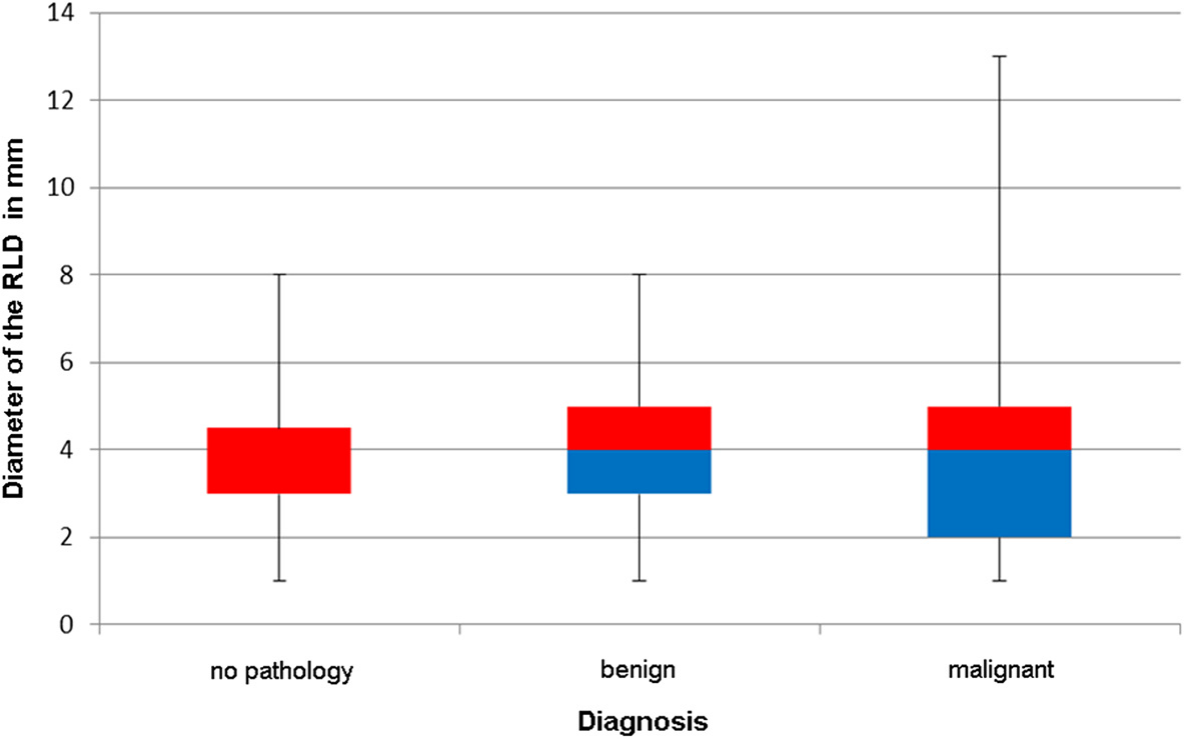
Box-Whisker-Plot for the diameter of confluence of the right lymphatic duct (RLD) depending on diagnosis

The mean diameter of the RLDs was 3.98 ± 1.96 mm. Males had a mean diameter of 4.08 ± 2.05 mm while the TD in women measured 3.71 ± 1.69 mm. The difference was not significant (*p* = 0.31). Healthy patients (group 1) had a mean diameter of 3.68 ± 1.7 mm, whereas it was 4.04 ± 1.64 mm in group 2 and 4.03 ± 2.13 mm in group 3 (Fig. 6). Taking into account only ipsilateral pathologies the mean diameter in group 2 was 4.16 ± 1.64 mm and 4.04 ± 2.11 mm in group 3. Neither of these differences were significant (*p* = 0.74 and *p* = 0.60).

**Fig. 7:**
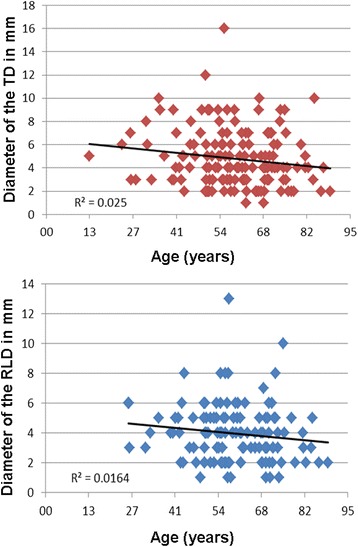
Diameters of the thoracic duct (TD) and right lymphatic duct (RLD) in millimeters (mm) in relation to patient‘s age in years

The mean diameter of the TD was 5.41 ± 2.55 mm in age group A whereas it was 4.6 ± 2.34 mm in age group B (*p* = 0.09). The values for the RLD were 4.07 ± 1.68 mm and 3.95 ± 2.04 mm respectively (*p* = 0.74). On the other hand a significant influence of age regarding the diameter of the TD could be determined in both the univariate (*p* = 0.0463) and multivariate (*p* = 0.0481) regression. This was not the case for the RLD. Figure 7 shows the relation of age and diameter for both the TD and the RLD. Contrary to this regarding healthy individuals (diagnosis group 1) a tendency for smaller diameters in younger individuals could be observed for the TD (4.44 ± 2.07 mm in age group A vs. 4.58 ± 3.11 mm in age group B) exlusively which was not the case for the RLD (4.13 ± 1.46 mm in age group A vs. 3.36 ± 1.86 mm in age group B). However both differences were not significant.

### Reflux of CM into the great lymphatic vessels

In 160 identified TDs a CM reflux could be observed in 40 cases (25 %). On the other hand in 120 identified RLDs a CM reflux was found in 21 patients (17.5 %). As the side of CM injection was not known, statistical analyses seemed not to be reasonable.

## Discussion

Altough the main lymphatic vessels (thoracic duct (TD) and right lymphatic duct (RLD)) in the cervical-thoracic junction are rather delicate anatomic structures we were able to identify them in the majority of cases even in routine computed tomography (CT) examinations of the head and neck region. The TDs' identification rate of 81.2 % in our study is quite higher than in previous reports about rates of 55 % and 63 % [1, 11]. This difference is even bigger concerning the identification of the RLD which was 64.2 % in our study and thus much higher than the 4 % in the report of Liu et al. [1]. As the diagnostic benefit of including coronal reformations into the reading of CT scans is well documented for other body regions [12–14], we conjecture that our higher detection rate can be explained by the fact that in our analysis also both the axial and coronal planes were included.

Moreover a difference in the slice thickness between 3 mm in our study and 1.5 mm from Gossner [11] could contribute to the different detection rates due to increased image noise in thinner sections. A detection frequency of 14 % for the distal TD in another study which used 1 mm slices may support this theory [15].

We categorized the morphology of th main lymphatic vessels into three groups (tubular, sacciform, dendritic). For the TD the distribution was tubular > sacciform > dendritic, even in most of the subgroups of patients. Our rate of 11.3 % of the dendritic type is comparable to Kinnaert’s analysis [16]. On the other hand the dendritic configuration was not described in other analyses: Liu et al. differentiated tubular (43 %), flared (45 %) and fusiform (12 %) subgroups [1]. Our higher (67.5 %) rate of tubular TDs may be explained by the fact, that in our analysis both healthy and sick patients were included whereas Liu et al. excluded patients with a history of cancer, presence of lymphadenopathy or neck abscesses. This could give rise to the assumption that unhealthy individuals tend to have tubular shaped TDs. However even in our “healthy” subgroup the tubular configuration could be observed in more than 6 out of 10 patients (64.3 %).

There seems to be an influence of patients' age on the distribution of configuration subgroups as the TD was described significantly more often as tubular or dendritic in the elderly (age group B) and as sacciform in the younger (age group A). However to find out if the morphology of the TD is subject to change during lifetime, patients would have to be observed in an additional study for a very long period.

The mean diameter of the TD varies between the reports in literature. While the mean diameter of healthy individuals in our study (4.54 mm) correlates quite well with the CT-based results of Liu et al. (4.8 mm [1]), lower mean diameters from 1.9 to 3.74 mm were reported for MRI [7, 9] and ultrasound [5, 17] (Table 1). This could be explained in some part by the different image resolution in different modalities and the possible influence of inspiration and expiration on the TD’s calibre [7]. On the other hand not all authors described in detail in what exact anatomic region the diameter of the TD was obtained. However according to Kiyonga et al. this seems to be of minor importance [15].

**Table 1:** Overview of mean imaging based diameters of the thoracic duct in literature

Authors	Imaging Modality	Mean Diameter	Range
Liu et al. [1]	CT	4.8 mm	–
Kiyonaga et al. [15]	CT	2.3 mm	1.1–2.8 mm
Yu et al. [9]	MRI	3.6 ± 0.1 mm	–
Takahashi et al. [7]	MRI	3.75 ± 0.81 mm	2.62–5.57 mm
Seeger et al. [5]	US	2.5 mm	–
Zironi et al. [17]	US	1.9 ± 0.5 mm	–
Parasher et al. [20]	US	2.43 mm	1.5–4 mm

*CT* computed tomography, *MRI* magnetic resonance imaging, *US* Ultrasound

As it is well documented in literature that pathologies of the body trunk may have an influence on the morphology of the TD (mostly diseases leading to increased hydrostatic pressure as portal hypertension or cardiac congestion) [2–9, 17] the central aim of the study was to investigate this phenomenon for the head and neck region for the first time. Theoretically there are two main causes for a dilatation of the main lymphatic vessels: overproduction or impeded drainage of lymph. Pathologies as inflammations or tumours often cause edema due to increased vessel permeability leading to a higher lymph volume. Moreover pathologies may induce both neohemangiogenesis and neolymphangiogenesis [18]. On the other hand besides compression or direct invasion tumours can also metastasize into the main lymphatic vessels [19] possibly leading to an obstruction of the duct. However in our study we did not find a significant influence of the presence of head and neck pathologies (including benign and malign tumours) on the morphology of the main lymphatic vessels, which correlates well with the results Takahashi et al. presented for malignancies of the body trunk [7].

While there exists quite a lot of literature about imaging of the TD, data about the RLD is very limited and makes comparison of our results almost impossible at the time of writing. We were able to detect the RLD less often than the TD (64.2 % vs. 81.2 %) but with a much higher frequency than Liu et al. reported (4 %, [1]). Analogical with the TD the discrepancy to the results of Liu et al. could be explained by us evaluating multiplanar images leading to a higher detection rate of this rather short anatomic structure. Similar to the findings on the left side we could not observe any significant correlation between the RLD’s detection rate or diameter and the presence of head and neck pathologies.

In our entire study population with increasing age a tendency of the diameter of the TD and RLD to decrease could be observed (diagrams 5, 6). Contrary to this regarding healthy individuals exclusively the diameter of the TD seems to increase with age, which correlates with the results of the healthy populations of other studies [1, 17]. The patients’ gender in our observation had no significant influence on detection rate or diameter of the main lymphatic vessels as reported before in CT or MRI studies [1, 9]. However Seeger et al. found a significant larger TD in males than in females using ultrasound [5].

As reported before reflux of highly concentrated contrast media from the veins into the RLD and TD can be observed in CT [1, 10]. We found that phenomenon quite more often than Liu et al. reported (21.8 % vs. 9 %), which again may be caused by including the coronal plane into our image evaluation. However as the identification of the injection side (right vs. left arm) was not possible in our retrospective study there seems to be no way of any reasonable statistical evaluation of this interesting finding.

Another limitation of the study lies within the lack of knowledge of individual factors (e.g. cardiac function, renal function, body height and weight) which could influence the detection rate, diameter and morphology of the main lymphatic vessels, too. Therefore our results should be correlated with further prospective evaluations including these informations.

## Conclusions

According to our results there is no influence of head and neck pathologies or age on the CT detection rate and morphology of the TD and RLD. On the other hand we could find a tendency of the ducts’ diameters to decrease with age. Among the various configurations of the distal TD and the RLD the tubular type predominates. In CT contrast media reflux into the main lymphatic ducts may be seen in more than one fifth of patients. Our study shows that both the RLD and the TD can be detected regularly in routine head and neck CT. Therefore reading physicians and radiologists should be familiar with its various imaging appearances to avoid mistaking these structures for a lesion and to be able to describe its precise location e.g. prior to surgery in order to prevent potential injury. Our higher detection rate of the main lymphatic vessels compared to previous work emphasizes the importance of the inclusion of multiplanar images in the evaluation of the quite complex anatomy of the head and neck region.
